# Development of a novel clinical decision support tool for diabetes prevention and feasibility of its implementation in primary care

**DOI:** 10.1016/j.pmedr.2022.101979

**Published:** 2022-09-07

**Authors:** Matthew J. O'Brien, Maria C. Vargas, Azucena Lopez, Yury Feliciano, Dyanna L. Gregory, Paula Carcamo, Loretta Mohr, Nivedita Mohanty, Roxane Padilla, Ronald T. Ackermann, Stephen D. Persell, Joseph Feinglass

**Affiliations:** a Division of General Internal Medicine and Geriatrics, Department of Medicine, Northwestern University Feinberg School of Medicine. 750 N. Lakeshore Drive, 10^th^ Floor, Chicago, IL 60611, United States; b Institute of Public Health and Medicine, Northwestern University Feinberg School of Medicine. 750 N. Lakeshore Drive, 6^th^ Floor, Chicago, IL 60611, United States; c Department of Preventive Medicine, Northwestern University Feinberg School of Medicine. 680 N. Lakeshore Drive, 14^th^ Floor, Chicago, IL 60611, United States; d Erie Family Health Centers. 1701 W. Superior Street, Chicago, IL 60622, United States; e AllianceChicago. 225 W. Illinois Street, 5^th^ Floor, Chicago, IL 60654, United States

**Keywords:** Prediabetes, Diabetes prevention, Primary care, Clinical decision support, Electronic health records

## Abstract

Prediabetes impacts 88 million U.S. adults, yet uptake of evidence-based treatment with intensive lifestyle interventions and metformin remains exceedingly low. After incorporating feedback from 15 primary care providers collected during semi-structured interviews, we developed a novel Prediabetes Clinical Decision Support (PreDM CDS) from August 2019 to February 2020. This tool included order options enabling prediabetes management in a single location within the electronic health record. We conducted a retrospective observational study examining the feasibility of implementing this tool at Erie Family Health Centers, a large community health center, examining its use and related outcomes among patients for whom it was used vs not. Overall, 7,424 eligible patients were seen during the implementation period (February 2020 to August 2021), and the PreDM CDS was used for 108 (1.5 %). Using the PreDM CDS was associated with higher rates of hemoglobin A1c orders (70.4 % vs 22.2 %; p < 0.001), lifestyle counseling (38.0 % vs 7.8 %; p < 0.001), and metformin prescription orders (5.6 % vs 2.6 %; p = 0.06). Exploratory analyses revealed small, nonsignificant weight loss among patients for whom the PreDM CDS was used. This study demonstrates the feasibility of developing and implementing the PreDM CDS in primary care. Its low use was likely related to not imposing an interruptive ‘pop-up’ alert, as well as major changes in workflows and clinical priorities during the Covid-19 pandemic. Use of the tool was associated with improved process outcomes. Future efforts with the PreDM CDS should follow standard CDS implementation processes that were not possible due to the Covid-19 pandemic.

## Highlights

• Clinical decision support may represent a strategy for promoting diabetes prevention in primary care.
• We developed a novel clinical decision support tool with input from primary care providers.
• This clinician-facing tool was associated with improvements in processes of prediabetes care.
• Exploratory analyses found small, but nonsignificant weight loss associated with its use.

## 1 Introduction

Recent studies estimate that 88 million American adults have prediabetes, up to 50 % of whom will develop type 2 diabetes (T2D) within 5 years. (Zhang et al., 2010, Centers for Disease Control and Prevention. National Diabetes Statistics Report, Estimates of Diabetes and Its Burden in the United States., 2022) However, a large body of research has demonstrated the effectiveness of interventions to prevent or delay T2D in this population. The landmark Diabetes Prevention Program trial randomized 3,234 adults with prediabetes to receive a structured intensive lifestyle intervention (ILI), metformin, or placebo. (Knowler et al., 2002) After 3 years, the reduction in T2D incidence associated with ILI and metformin was 58 % and 31 %, respectively; (Knowler et al., 2002) and weight loss was the dominant predictor of reduced T2D risk with both treatments. (Hamman et al., 2006) While many subsequent trials of ILI and metformin have demonstrated similar effectiveness in real-world settings, (Ali et al., 2012, Dunkley et al., 2014, Mudaliar et al., 2016) these treatments are used by<1 % of U.S. adults with prediabetes. (Tseng et al., 2017, YMCA's Diabetes Prevention Program, 2018, Gruss et al., 2019) This represents a significant gap in translating evidence about diabetes prevention into practice.

With 440 million primary care visits made by U.S. adults annually, (Santo and Okeyode, 2018) this represents an important venue for promoting diabetes prevention. Prediabetes is most commonly diagnosed in primary care, creating natural opportunities to offer evidence-based treatment. (American Diabetes Association, 2015) However, only 15.3 % of adults with prediabetes report being told by a healthcare provider about having the condition, (Centers for Disease Control and Prevention. National Diabetes Statistics Report, Estimates of Diabetes and Its Burden in the United States., 2022) and even fewer have been linked to evidence-based preventive treatments. Clinical decision support (CDS) uses electronic systems to aid in clinical decision making, using individual patient data to generate tailored recommendations that are presented to clinicians. (Kawamoto et al., 2005) CDS has been extensively applied to the care of patients with T2D, with many studies reporting greater adherence to evidence-based clinical services and improved glycemic outcomes. (O'Connor et al., 2016) To our knowledge, only one prior study has evaluated the application of CDS for managing prediabetes, and another is currently collecting outcome data. (Mann et al., 2016, Desai et al., 2022).

Given the paucity of prior work in this area, the current study objectives were to: 1) interview providers about their preferences for CDS focused on prediabetes; 2) develop a novel CDS tool, the Prediabetes CDS (PreDM CDS), promoting evidence-based care for prediabetes; and 3) conduct a pilot evaluation of the novel CDS tool using electronic health record data.

## 2 Materials and methods

### 2.1 PreDM CDS development

We designed the PreDM CDS with AllianceChicago, a Health Center Controlled Network that provides health information technology infrastructure to Erie Family Health Centers (Erie), including a clinical data warehouse and an electronic health record (EHR) system on the GE Centricity platform. Erie is a large federally funded community health center that serves a predominantly Hispanic/Latino patient population and was the clinical partner for this study. The investigative team, composed of prediabetes experts, clinical informaticists, primary care clinicians and study staff, met regularly from August 2019 to February 2020 to develop the PreDM CDS, making iterative changes to its design and functions.

To help guide the design of the clinician-facing PreDM CDS, we conducted semi-structured individual interviews with 15 primary care providers at Erie. Participating providers were recruited by the project lead at Erie (L.M.), who was also a primary care provider there. Our interview guide was designed to solicit providers’ preferences for CDS design features that would facilitate evidence-based prediabetes care. The interview guide was structured according to the 5 Rights Framework, a widely accepted model for developing CDS interventions. (Osheroff, 2009) Semi-structured provider interviews were conducted by a research coordinator and were recorded for qualitative analysis, which followed methods described in the Rapid Identification of Themes from Audio Recordings. (Neal et al., 2015).

### 2.2 PreDM CDS design and functions

This CDS tool was intended for clinicians’ use with adult patients aged ≥ 18 years who have prediabetes. It was designed to appear automatically only for patients with this condition during both in-person and telemedicine visits. The EHR-based algorithm for displaying the PreDM CDS defined prediabetes by the presence of a diagnosis code for prediabetes or available glycemic test results in the prediabetes range. This algorithm excluded patients with active pregnancy or diabetes, as evidenced by prior glycemic test results in the diabetes range, diabetes diagnosis codes documented in the EHR, or antidiabetic medication orders. These criteria for defining diabetes and prediabetes are displayed fully in Appendix A. The algorithm also excluded patients with the last creatinine value > 1.4 mg/dL in women and > 1.5 mg/dL in men because some prescribing guidelines recommend avoiding metformin above these cutoffs.

**Fig. 1:**
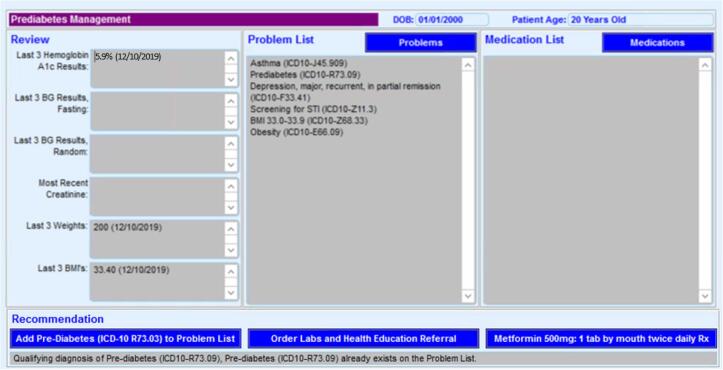

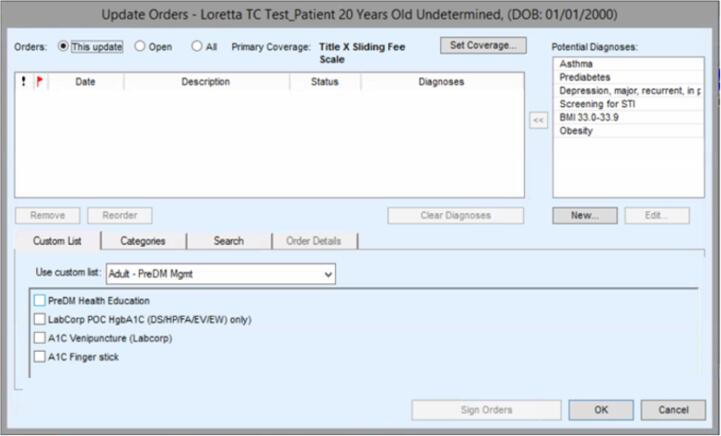
1a PreDM CDS Display of Relevant Data and Functions for Prediabetes Management 1b PreDM CDS Options for Placing A1c Orders and Health Education Referrals.

Images of the PreDM CDS are shown in Fig. 1. The PreDM CDS is a passive EHR button that appears automatically under the Assessment/Plan only for patients with prediabetes, rather than an interruptive ‘pop-up’ alert requiring clinicians to click on the tool. When clinicians choose to click on this button, the PreDM CDS displays the last three measurements of weight, body mass index (BMI), hemoglobin A1c (HbA1c), fasting glucose, random glucose, and creatinine (Fig. 1a). The latter lab value was included to inform decisions about prescribing metformin safely. Below this display, providers can select any of the following functions that were included in the PreDM CDS based on review of existing literature, expert opinions by study team members, and provider feedback: 1) add a prediabetes diagnosis code to the problem list; 2) prescribe metformin; 3) order A1c for patients without a recent measurement; and 4) refer patients to a health educator for counseling about healthy lifestyle change and Erie’s intensive lifestyle intervention (ILI) based on the Diabetes Prevention Program (Fig. 1a). Prior research has found increased engagement in ILI if participants receive counseling about the program before enrolling. (Ritchie et al., 2018) The latter two functions are enabled by clicking a button entitled “Order Labs and Health Education Referral,” which links to the menu where these orders are placed (Fig. 1b).

### 2.3 Implementation of the PreDM CDS and related context

PreDM CDS was deployed in the EHR as planned on February 26, 2020, approximately-one week before the Covid-19 pandemic led to a statewide stay-at-home order and caused substantial disruptions in patient care, workflows, and clinical priorities at Erie. Throughout the study period, there were many changes in provider demands and workflows related to the evolving Covid-19 pandemic and its impact on clinical care. As a result, Erie could not follow its usual process for implementing the PreDM CDS that includes regular provider trainings, technical assistance, and reminders. When the PreDM CDS was launched, all providers at the 14 participating clinic sites received an email describing the tool with embedded screenshots and a brief video displaying its functions.

### 2.4 Evaluation of the PreDM CDS

#### 2.4.1 Study design, setting and eligibility

We conducted a retrospective, observational cohort study of the PreDM CDS implementation at Erie from February 26, 2020 to August 1, 2021. We analyzed retrospective EHR data collected during routine primary care encounters of patients with the following characteristics for whom the PreDM CDS appeared: age ≥ 18 years; prediabetes; and ≥ 2 wt measurements during the study period. The latter inclusion criterion enabled an exploratory analysis of weight change during the study period (Section 2.4.2). We excluded from the EHR cohort patients who had: evidence of diabetes; active pregnancy; or elevated creatinine level that could preclude metformin use (i.e., >1.4 mg/dL in women and > 1.5 mg/dL in men). (Lipska et al., 2011) The index date for each patient was the date of their first clinical measurement after the PreDM CDS launch, which served as the baseline value and the assessment of CDS use. Their final clinical measurement as of August 1, 2021—and occurring at least three months after the index date—served as the follow-up value. Because using EHR data to direct deployment of the PreDM CDS imposed no additional risk to eligible patients, the study was conducted under a waiver of written informed consent. The study protocol was approved by the Northwestern University Institutional Review Board.

#### 2.4.2 Covariates and study outcomes

We examined the following patient demographic characteristics: age, sex, race/ethnicity, and insurance status. In addition, we assessed the presence of the following clinical risk factors for developing diabetes: overweight/obesity, dyslipidemia, family history of diabetes, and gestational diabetes in women. Definitions of these covariates are displayed in Appendix A.

We studied several process outcomes related to use of the PreDM CDS. Specifically, we assessed the proportion of eligible patients for whom this tool was used, including each of its linked functions (i.e., adding prediabetes diagnosis code, ordering an A1c test, prescribing metformin, and placing a health educator referral). Of those who attended a health educator counseling session about prediabetes, we observed the proportion of patients who subsequently attended at least one ILI session. We also examined use of the PreDM CDS by individual providers, provider type (nurse practitioner or physician), provider specialty, and clinic site.

We conducted an exploratory analysis of weight change related to PreDM CDS use, which was assessed by the difference between the first and last weight measurement during the study period, requiring a minimum of 90 days between measurements. Continuous weight change was used to create a dichotomous outcome for losing at least 2.2lbs, which we adopted as a minimally important difference because it is associated with a 16 % reduction in diabetes incidence. (Hamman et al., 2006).

#### 2.4.3 Statistical analysis

Descriptive statistics were used to assess participants’ baseline characteristics and use of the PreDM CDS during the study period. The significance of differences in baseline characteristics among participants for whom the PreDM CDS was used vs not used was examined using chi-square tests for categorical variables and t-tests for continuous variables. Due to small numbers of patients with gestational diabetes and a family history of diabetes, Fisher’s exact test was used to assess the difference in those risk factors among participants for whom the PreDM CDS was used vs not used. Continuous change in weight was examined in an exploratory linear regression model adjusted for age, sex, race/ethnicity, baseline weight, and time between the baseline and follow-up weight measurements. A p-value of < 0.05 was considered significant for all statistical testing. All analyses were conducted using SAS, version 9.4.

## 3 Results

### 3.1 Findings from clinician interviews used to inform PreDM CDS development

Most providers expressed a preference for not using ‘pop-up’ alerts in the PreDM CDS that would require their response. Providers mentioned that there are already many such clinical alerts in their EHR, which they deemed intrusive. Many interviewees mentioned that they regularly circumvent such alerts to continue patient care activities without interruption. Providers wanted the PreDM CDS to include a display of recent weights and glycemic measurements, which would help them decide which orders to place and assist in related patient counseling efforts. Providers consistently recommended that the PreDM CDS also include a bundled order set with all options for evidence-based prediabetes management in a single location. This would facilitate ordering each of these functions quickly, given that their prior workflow required accessing multiple EHR locations to place the same orders. The specific orders suggested by providers to promote evidence-based prediabetes care were ultimately included (Section 2.2 above). The metformin dose included in the PreDM CDS order function (i.e., 500 mg twice daily) was decided by consensus among providers interviewed and study team experts on diabetes prevention. Representative quotes from providers supporting their preferences for these PreDM CDS design features are included in Table 1.

**Table 1:** Selected Qualitative Feedback from Primary Care Providers about Design of the PreDM CDS.

PreDM CDS Design Features	Representative Quotes
No pop-up alert prompts	“Sending an alert is not a solution because I can easily close the alert and say I have a lot of things to do.“ “I feel like we get bombarded with alerts…but I think even with that we somehow miss [them]. I do not know if it is because we get too many of them or they come in different formats [that] they do not become what they are meant to be used as.”
Display of biomarker data	“Providers would appreciate an A1c trend like they are able to see for blood pressure. All providers typically use the [vital sign] landing page at every visit, which allows for a quick vital signs review.” “I think it's helpful to show patients where they are and what their [weight] goal is. To see where they are trending is very helpful.”
Bundled order set	“Automating as much as we can is a good thing. Order sets are great! Order sets are bundles of the right care so we do not miss things. Absolutely, we just don't have that. Bundle sets are good. Epic has bundle sets-it makes sure I do not miss things.” “The more the computer can do for you the better. You make a click, and the EHR can generate an order set, help with referral and flag health educator that would facilitate either referring patients to lifestyle programs or metformin prescription.”

### 3.2 Characteristics of patients included in the pilot study and their providers

We analyzed data on a cohort of 7,424 patients, who met eligibility criteria listed above (Section 2.4.1) and for whom the passive PreDM CDS appeared in the EHR during their office visits over the study period, giving their providers the opportunity to use the CDS. Overall, the PreDM CDS was used by providers caring for 108 of these patients (1.5 %). Only 14 PreDM CDS uses (13 %) occurred during telemedicine visits. In total, 27 of 176 providers (15.3 %) used the PreDM CDS, many of whom (70 %) used it only 1 or 2 times. Over half of users were nurse practitioners, and more uses were attributed to family practitioners than providers from any other clinical specialty. Almost 60 percent of PreDM CDS uses occurred at a single clinic site, which has been most engaged in prior related research efforts. (Table 2).

**Table 2:** Attribution of PreDM CDS Usage by Provider Characteristics and Clinic Site.

Attribution of PreDM CDS Use	N (%)
Total Uses^a^	104 (1 0 0)
By Individual Provider^b^	
Number of providers who used PreDM CDS	27 (1 0 0)
Number of providers with 1–2 uses	19 (70)
Number of providers with 3–5 uses	6 (22)
Number of providers with ≥ 6 uses	2 (7)
By Provider Type	
Number of uses by nurse practitioners	61 (59)
Number of uses by physicians	43 (41)
By Provider Clinical Specialty	
Family medicine	42 (40)
Internal medicine	31 (30)
Women’s health	18 (17)
Unknown	13 (12)
By Clinic Site	
Number of uses at clinic site 1	61 (59)
Number of uses at clinic site 2	14 (13)
Number of uses at clinic site 3	9 (9)
Number of uses at clinic site 4–14	20 (19)

a The attribution of PreDM CDS use by provider and clinic site was not available for 4 instances (4%) of its use. Therefore, the total number of PreDM CDS uses included in this table is 104, which serves as the denominator for the percentages reported.

b The number of unique providers who used the PreDM CDS was 27 out of a total of 176 providers (15.3%). The 27 providers who used the CDS served as the denominator for the reported percentages on individual provider use.

The mean follow-up time was 148.9 days. Most patients in the cohort were aged 35–64 years with Hispanic/Latino ethnicity and a significant burden of cardiometabolic risk factors, including prediabetes (100 %), hypertension (36 %) and dyslipidemia (35 %). Use of the PreDM CDS was more common among those aged 35–50 years old, with a family history of diabetes, women, and Hispanics/Latinos. In bivariate analyses, PreDM CDS use was significantly associated with female sex and a family history of diabetes. (Table 3).

**Table 3:** Baseline Participant Characteristics by PreDM CDS Use^a^.

Characteristic	CDS Not Used,N (%)	CDS Used,N (%)	P-value^b^
Number of Participants	7316 (98.5)	108 (1.5)	
Age, years			0.87
18–34	1456 (19.9)	20 (18.5)	
35–50	3217 (44.0)	50 (46.3)	
51–64	2105 (28.8)	32 (29.6)	
≥65	538 (7.4)	6 (5.6)	
Sex			0.02
Female	5002 (68.4)	85 (78.7)	
Male	2308 (31.6)	23 (21.3)	
Unknown	6 (0.0)	0 (0.0)	
Race/ethnicity			0.35
White	406 (5.6)	3 (2.8)	
Black	612 (8.4)	8 (7.0)	
Hispanic/Latino	5758 (78.7)	93 (86.1)	
Other/Unknown	540 (7.4)	4 (3.7)	
Insurance Status			0.86
Private	872 (11.9)	14 (13.0)	
Medicaid	2296 (31.4)	37 (34.3)	
Medicare	193 (2.6)	4 (3.7)	
Uninsured	3800 (52.0)	51 (47.2)	
Other/Unknown	155 (2.1)	2 (1.8)	
Body mass index,^c^ kg/m^2^	33.1 (6.6)	33.4 (6.5)	0.57
Hemoglobin A1c,^c^ %	5.8 (0.3)	5.8 (0.3)	0.38
Hypertension^d^	2628 (35.9)	35 (32.4)	0.45
Dyslipidemia^d^	2581 (35.2)	32 (29.6)	0.22
History of gestational diabetes^d,e^	74 (1.0)	0 (0.0)	0.63
Family history of diabetes^d,e^	109 (1.5)	6 (5.6)	<0.001

a Baseline characteristics were assessed for all participants meeting eligibility criteria, and stratified by whether the PreDM CDS was used during the study period.

b P-values were derived from chi-square tests and t-tests examining the significance of differences in baseline characteristics between participants for whom the PreDM CDS was used vs those for whom it was not.

c Variable is expressed as mean (SD).

d Definitions of diabetes risk factors are included in Appendix A.

e Due to small numbers for gestational diabetes and family history of diabetes, Fisher’s exact test was used to assess the significance of differences in these risk factors between groups.

### 3.3 Outcomes related to PreDM CDS use

Patients for whom the PreDM CDS was used exhibited significantly higher rates of ordering HbA1c lab tests (70.4 % vs 22.2 %; p < 0.001) than those for whom the CDS was not used. The mean time between A1c orders placed during the study period and patients’ previous A1c result was 385 days. We also observed higher rates of referring patients to a health educator for counseling (34.3 % vs 6.9 %; p < 0.001) and attending health educator counseling about ILI (38.0 % vs 7.8 %; p < 0.001). Among the 41 patients for whom the PreDM CDS was used and subsequently attended lifestyle counseling, 37 (90.2 %) were referred to the health educator through the CDS and the other 4 patients (9.8 %) were referred through routine workflows outside the CDS. We also observed a higher rate of metformin prescription orders among those for whom the PreDM CDS was used, which did not achieve statistical significance (5.6 % vs 2.6 %; p = 0.06). Only five participants in the entire cohort attended an ILI session (0.001 %), none of whom had the PreDM CDS tool used by their providers beforehand. There was no significant difference in the proportion of patients who lost 2.2 lb (26.9 % among those in the PreDM CDS group vs 23.6 % of others; p = 0.43). (Table 4) In an exploratory multivariable analysis, there was no significant difference in weight loss observed between the groups for whom the PreDM CDS was used vs not used (-0.4 lb; 95 % CI: −1.9, 1.2). The weight loss observed among 41 CDS patients who attended health educator counseling was −0.7 lb (±6.5 lb).

**Table 4:** Dichotomous Outcomes According to PreDM CDS Use.

Outcomes^a^	CDS Not Used	CDS Used	P-value^b^
Number of participants	7316	108	N/A
Process Outcomes			
Prediabetes diagnosis code	1055 (14.4)	14 (13.0)	0.67
Hemoglobin A1c order	1623 (22.2)	76 (70.4)	<0.001
Metformin prescription	191 (2.6)	6 (5.6)	0.06
Referral to health educator	504 (6.9)	37 (34.3)	<0.001
Attended health educator counseling about diabetes prevention	569 (7.8)	41 (38.0)	<0.001
Attended any ILI session^c^	5 (0.1)	0 (0.0)	1.00
Clinical Outcome			
Lost ≥ 2.2 lb	1726 (23.6)	29 (26.9)	0.43

ILI = Intensive lifestyle intervention based on the Diabetes Prevention Program.

a Outcomes are reported as n (%).

b P-values were derived from chi-square tests examining the significance of differences in outcomes between participants for whom the PreDM CDS was used vs not used.

c Fisher’s exact test was used to test the significance of group differences in this outcome.

## 4 Discussion

We developed the novel PreDM CDS intervention promoting evidence-based prediabetes care and demonstrated the feasibility of its implementation in a pilot study. Use of this CDS tool was associated with significant increases in ordering HbA1c tests and referring patients for counseling about intensive lifestyle intervention (ILI). Further, we observed a greater than twofold increase in metformin prescriptions among those for whom the PreDM CDS was used vs not used. These findings show promise that CDS, aligned with clinicians’ preferences, can help improve the management of prediabetes in a busy primary care setting. Importantly, our CDS innovation was developed and implemented in a safety-net community health center, where historically underserved patients have particularly high risk of developing diabetes and often few available resources for prevention. (Hill et al., 2013, Hill-Briggs et al., 2021).

By focusing on prediabetes management, the novel PreDM CDS addresses an important and challenging clinical area where uptake of evidence-based treatments is vanishingly low. This pilot study was based in primary care clinics, which represent a promising venue for diabetes prevention efforts given their broad reach and the frequent identification of prediabetes in this setting. However, little prior research promoting ILI and metformin for adults with prediabetes has been conducted in primary care. Guided by input from primary care providers, the PreDM CDS includes a number of order options that support evidence-based prediabetes care. Allowing providers to quickly document prediabetes diagnosis codes and order HbA1c testing in the same EHR location as ordering metformin prescriptions and ILI referrals has the potential to improve population health management for prediabetes by simultaneously enabling surveillance and treatment.

Our pilot study also has notable limitations. Most significantly, the PreDM CDS was launched at the end of February 2020, only one week before widespread containment measures to mitigate the spread of Covid-19 were implemented. This timing made it impossible to conduct provider training and technical assistance used routinely for implementing new CDS tools. Our clinical partner for this study also closed many of its clinic sites in early March 2020, while dedicating some clinics to seeing only patients with symptoms potentially related to Covid-19 infection. Even among clinic sites that remained open for routine primary care, patient volume was significantly reduced and the management of early-stage cardiometabolic conditions like prediabetes was not a top priority. Disruptions in clinical workflows due to the Covid-19 pandemic not only impacted use of our novel CDS intervention by providers, but also restricted the availability of ILI programs at our clinic partner during the study period.

While these significant Covid-related challenges hindered our ability to study the clinical effectiveness of the PreDM CDS, other potential reasons for low uptake of the PreDM CDS should also be investigated. In addition, the lack of a randomized control group limits causal inference about whether the observed outcomes resulted directly from the PreDM CDS. Future studies with the PreDM CDS should follow ‘best practices’ for CDS implementation and use a randomized design to evaluate the same process and clinical outcomes definitively.

Only one prior study has evaluated a CDS intervention intended for use among primary care patients with prediabetes, (Mann et al., 2016, Mann and Lin, 2012) which prompted provider counseling to set specific dietary and physical activity goals with patients. During subsequent visits, providers could track patients’ progress at achieving those goals through the CDS tool. This pilot study demonstrated a significant increase in daily step counts among the 27 patients randomized to receive the CDS vs 27 patients who received usual care (+1,418 steps vs −598 steps respectively, p = 0.01). There were no significant differences reported for cardiometabolic markers including weight, HbA1c, or lipid values. (Mann et al., 2016).

This prior CDS tool prompted provider counseling efforts using interruptive ‘pop-up’ alerts, which prior studies have found to be burdensome and are therefore frequently overridden by providers. (Cash, 2009) In addition, prior research demonstrates that primary care providers counsel patients with prediabetes about healthy lifestyle change<30 % of the time. (Wu et al., 2019) These data suggest that CDS interventions aimed at promoting in-depth counseling by primary care providers may not be scalable or sustainable. Finally, this earlier pilot trial was limited by the small number of participants.

Our PreDM CDS offered primary care providers a list of actions for managing prediabetes that they suggested during interviews conducted as part of this tool’s development. While avoiding EHR ‘pop-up’ alerts was responsive to providers’ preferences and intended to avoid potential unintended consequences from forced EHR functions, (Sittig et al., 2008, Bisantz and Wears, 2009) this voluntary approach for using our PreDM CDS was partly responsible for its low uptake in this pilot study. Strategies to increase its use could include provider training, technical assistance, and reminders, as well as identifying a provider ‘champion’ at each clinic site who could provide ongoing guidance to other providers about its use. Our PreDM CDS attempted to shift the task of lifestyle counseling from primary care providers to health educators, for whom this activity falls directly under their scope of practice. While task shifting represents one strategy for overcoming barriers to provider counseling efforts, lifestyle counseling by health educators was performed for less than half of the CDS participants in the current study (38 %). This was likely related to workflow challenges imposed by needing to schedule a separate health education visit and exacerbated by many workflow changes stemming from the Covid-19 pandemic.

Using health educators to conduct lifestyle counseling may be limited to primary care offices that employ these professionals. However, a similar health education function is usually performed by other clinical staff members in primary care, including nurses, dieticians, or diabetes educators. Unfortunately, no patients who attended health educator counseling joined ILI. Overall, ILI attendance among the entire cohort with prediabetes was very low (0.001 %), which was partly related to limited ILI availability during the pandemic study period. However, it is estimated that only 143,489 adults have participated in ILI nationwide, representing a comparable rate of ILI attendance among the 88 million U.S. adults with prediabetes (i.e., 0.002 %). (Centers for Disease Control and Prevention. National Diabetes Statistics Report, Estimates of Diabetes and Its Burden in the United States., 2022, Gruss et al., 2019).

In exploratory analyses, we observed a nonsignificant difference in weight loss among patients for whom the CDS was used vs not used. This small improvement may have been related to lifestyle counseling conducted by health educators, which was completed by 38 % of patients in the CDS group and associated with greater weight loss.

## 5 Conclusions

Our study demonstrated the feasibility of developing and implementing the novel PreDM CDS, while finding improvements in processes of prediabetes care. Our study observed no significant differences in ILI participation or weight change among patients for whom the PreDM CDS was used. Because these are the primary intended outcomes of encouraging clinicians to offer evidence-based prediabetes treatment, future research designed to strengthen linkages to and persistent engagement in effective ILI programs should remain a top priority.

## Credit authorship contribution statement

**Matthew J. O'Brien:** Conceptualization, Methodology, Validation, Investigation, Resources, Writing – original draft, Writing – review & editing, Supervision, Funding acquisition. **Maria C. Vargas:** Methodology, Investigation, Writing – review & editing, Project administration. **Azucena Lopez:** Methodology, Writing – review & editing. **Yury Feliciano:** Methodology, Writing – review & editing. **Dyanna L. Gregory:** Software, Validation, Formal analysis, Data curation, Writing – review & editing, Visualization. **Paula Carcamo:** Methodology, Writing – review & editing, Project administration. **Loretta Mohr:** Methodology, Validation, Investigation, Writing – review & editing, Supervision. **Nivedita Mohanty:** Conceptualization, Methodology, Software, Validation, Resources, Writing – review & editing, Supervision. **Roxane Padilla:** Methodology, Validation, Writing – review & editing, Project administration. **Ronald T. Ackermann:** Conceptualization, Methodology, Writing – review & editing. **Stephen D. Persell:** Methodology, Writing – review & editing. **Joseph Feinglass:** Conceptualization, Methodology, Writing – review & editing.

## Declaration of Competing Interest

The authors declare that they have no known competing financial interests or personal relationships that could have appeared to influence the work reported in this paper.

## Figures and Tables

**Table:** 

**Covariate**	**ICD-10 Codes**^b^	**Laboratory Values or Clinical Measurements**^c^	**Medication prescriptions**
Diabetes	E10.x, E11.x, E13.x	Hemoglobin A1c ≥ 6.5 %Glucose: fasting (≥126 mg/dL),random (≥200 mg/dL),2-hour (≥200 mg/dL)	Antidiabetic medication
Prediabetes	R73.x	Hemoglobin A1C 5.7–6.4 % Fasting glucose: 100–125 mg/dL2-hour glucose: (140–199 mg/dL)	No antidiabetic medication
Overweight/obesity	Z68.25-Z68.45, E66.x	Body mass index ≥ 25 kg/m^2^	N/A
Hypertension	I10.x, I11.x, I12.x, I13.x, I15.x, I16.x	Blood pressure > 140/90 mmHg	Antihypertensive medication
Dyslipidemia	E78.0, E78.1, E78.2, E78.3, E78.4, E78.5	HDL cholesterol < 35 mg/dL Triglycerides > 250 mg/dL	Lipid-lowering medication
History of gestational diabetes	O24.4x, Z86.32	N/A	N/A
Family history of diabetes	Z83.3	N/A	N/A

## Data Availability

Data will be made available on request.

## Appendix A

Electronic Health Record Definitions of Diabetes, Prediabetes, and Related Clinical Risk Factors^a^.a In order to be classified as having any of the listed conditions or risk factors, patients needed evidence of at least 1 of the criteria presented.

b Diagnostic codes are based on the International Classification of Diseases, Tenth Revision (ICD-10).

c Diagnostic ranges for laboratory values and clinical measurements were derived from the American Diabetes Association’s 2021 Standards of Care.
